# Malaria Parasitemia and Nutritional Status during the Low Transmission Season in the Presence of Azithromycin Distribution among Preschool Children in Niger

**DOI:** 10.4269/ajtmh.19-0547

**Published:** 2020-02-17

**Authors:** Ahmed M. Arzika, Ramatou Maliki, Nameywa Boubacar, Salissou Kane, Catherine A. Cook, Elodie Lebas, Ying Lin, Kieran S. O’Brien, Ariana Austin, Jeremy D. Keenan, Thomas M. Lietman, Catherine E. Oldenburg, for the MORDOR Study Group

**Affiliations:** 1 The Carter Center Niger, Niamey, Niger;; 2 Francis I Proctor Foundation, University of California, San Francisco, San Francisco, California;; 3 Department of Ophthalmology, University of California, San Francisco, San Francisco, California;; 4 Department of Epidemiology & Biostatistics, University of California, San Francisco, San Francisco, California

## Abstract

The relationship between malaria and malnutrition is complicated, and existence of one may predispose or exacerbate the other. We evaluated the relationship between malaria parasitemia and nutritional status in children living in communities participating in a cluster-randomized trial of biannual azithromycin compared with placebo for prevention of childhood mortality. Data were collected during the low malaria transmission and low food insecurity season. Parasitemia was not associated with weight-for-height *Z*-score (24 months: *P* = 0.11 azithromycin communities, *P* = 0.75 placebo communities), weight-for-age *Z*-score (24 months: *P* = 0.83 azithromycin, *P* = 0.78 placebo), height-for-age *Z*-score (24 months: *P* = 0.30 azithromycin, *P* = 0.87 placebo), or mid-upper arm circumference (24 months: *P* = 0.12 azithromycin, *P* = 0.56 placebo). There was no statistically significant evidence of a difference in the relationship in communities receiving azithromycin or placebo. During the low transmission season, there was no evidence that malaria parasitemia and impaired nutritional status co-occur in children.

## INTRODUCTION

In Niger, the high malaria transmission season overlaps with periods of high food insecurity and malnutrition.^1–3^ Despite this seasonal overlap, the relationship between malaria and malnutrition at the individual level is complicated.^4^ Previous studies in a variety of different geographic settings have yielded mixed results, with no consistent association between malaria and acute malnutrition and some evidence that chronic malnutrition is associated with malaria severity.^4^ A previous study in Niger conducted at the beginning of the malaria season found evidence of a small increase in malaria parasitemia with fever in children with higher height-for-age *Z*-scores (HAZ), but no other evidence of an association between nutritional status and malaria.^5^ A systematic review of studies of the relationship between malaria and malnutrition concluded that the relationship is complex and called for analysis of additional individual patient data of malaria and malnutrition in different settings.^4^ Poor nutritional status may predispose children to infection with malaria by modulating immune function, and children with malaria may be predisposed to malnutrition, particularly if they suffer from repeated infection. Conversely, some studies have shown a protective effect of malnutrition against malaria, which may be mediated by iron or other nutritional deficiencies.^6^ Given the complexity of any potential relationship, it is possible that the relationship may differ in different geographic or seasonal settings or in the presence of interventions that affect one or both conditions.

Azithromycin has modest activity against malaria by targeting the plasmodial apicoplast.^7,8^ Although studies of mass azithromycin distribution for trachoma control have yielded mixed results,^9–12^ a study of biannual mass azithromycin distribution for child mortality demonstrated a significant decrease in malaria parasitemia in communities receiving azithromycin compared with those receiving placebo.^13^ Any relationship between malaria and malnutrition could potentially be affected by distribution of azithromycin to children given the overall effect in a reduction in malaria parasitemia.

Here, we investigated the relationship between malaria parasitemia and malnutrition in the same communities in which azithromycin was shown to decrease malaria parasitemia in Niger compared with biannual placebo to assess the relationship between parasitemia and nutritional status in the low transmission season and any effect of the azithromycin intervention on that relationship.

## METHODS

Data arose from the Macrolides Oraux pour Réduire les Décès avec un Oeil sur la Résistance (MORDOR) study, a cluster-randomized trial in which communities in Boboye and Loga, Niger, were randomized in a 1:1 fashion to either biannual azithromycin distribution or placebo to children aged 1–59 months (clinicaltrials.gov NCT02048007).^14^ No mass azithromycin distributions for trachoma occurred within 5 years before the study, and the area was not receiving seasonal malaria chemoprevention at the time of the study. Thirty communities were randomly selected from the same pool as the main MORDOR study for more intensive monitoring and similarly randomized to biannual distributions of azithromycin or placebo. After the biannual census and before treatment, children were monitored with anthropometric assessments and blood smears for malaria parasitemia. Ethical approval was obtained from the Committee on Human Research at the University of California, San Francisco, and the Institutional Review Board of the Nigerien Ministry of Health.

Communities were randomized at the level of the grappe, a government-defined health catchment area, henceforth referred to as “community.” Communities were eligible if they had a population size between 200 and 2,000 individuals on the most recent government census before the study. All households in each census were enumerated approximately every 6 months in a house-to-house census.

A random sample of 40 children aged 1–59 months per community were randomly selected from the census for monitoring. Children were monitored in March 2015, June 2016, and April 2017. The 12-month monitoring visit occurred after two rounds of study treatment and the 24-month visit occurred after four rounds of study treatment. Each child had a finger stick to estimate hemoglobin with HemoCue Hb 201^+^ (HemoCue, Ängelholm, Sweden) and thin and thick smears for malaria parasitemia. Malaria parasitemia was considered the presence of malaria parasites on thick smear. Laboratory personnel were masked to treatment arm. Anthropometric measurements, including weight, height, and mid-upper arm circumference (MUAC) were measured in all children. Children were weighed standing if able or in the arms of a caregiver (Seca 874 flat floor scale, Seca GMBH & Co., Hamburg, Germany), with heavy garments and jewelry removed before weight measurements. Recumbent length was measured in children who were not able to stand and standing height in children who could stand (Shorrboard, Shorr Products, LLC). Weight and height were measured in triplicate, and the median was used for analysis. Weight-for-height *Z*-scores (WHZ), HAZ, and weight-for-age *Z*-scores (WAZ) were calculated based on 2006 WHO standards.^15^ Wasting, underweight, and stunting were defined as WHZ, WAZ, and HAZ scores of < −2 SD, respectively.

Children were offered treatment if they were between 1 and 59 months of age and weighed at least 3,800 g. A single directly observed oral dose of study drug (azithromycin 20 mg/kg or matching placebo) was administered to all eligible children approximately every 6 months. Children were offered treatment after all monitoring activities were completed. Dosing was calculated based on height-stick approximation for children who could stand or by weight for children who were unable to stand.^16^

We used mixed effects linear and logistic regression models to estimate the relationship between malaria parasitemia and nutritional status. Because of the cross-sectional nature of the data, the goal of this analysis was to estimate associations between parasitemia and nutritional status rather than estimate causal effects. Children who had valid anthropometric and parasitemia measurements were included in analyses. Because children aged 1–59 months were randomly sampled at each time point, we did not have longitudinal measurements for most children. Furthermore, because samples were collected in 1-year intervals, we felt that any relationship between malaria parasitemia and nutritional status measurements would be unlikely to be correlated over such a long time period. For these reasons, all analyses were cross-sectional analyses conducted at each sample collection time point (e.g., baseline, after 12 months/two study treatments and after 24 months/four study treatments). A separate model was built for each time point. Models were built predicting nutritional status indicators (WHZ, HAZ, WAZ, and MUAC) as continuous variables and dichotomized as wasting (WHZ < −2 SD), stunting (HAZ < −2 SD), and underweight (WAZ < −2 SD). Models included fixed effects for malaria parasitemia status, the child’s age and gender, community treatment assignment, an interaction term between treatment assignment and malaria parasitemia, and a random effect for study community. All analyses were conducted in R version 3.5.3 (The R Foundation for Statistical Computing, Vienna, Austria).

## RESULTS

Baseline characteristics by malaria parasitemia status are summarized in Table 1. Prevalence of parasitemia at baseline was 7.9%. Most baseline characteristics were similar between children with malaria parasitemia and those without (Table 1), although children with malaria parasitemia had lower hemoglobin levels (9.35 g/dL versus 9.9 g/dL, *P* = 0.0006) and lower WHZ and HAZ scores, although these were not statistically significant (Table 2).

**Table 1:** Descriptive characteristics by malaria parasitemia at baseline

	Malaria parasitemia	No malaria parasitemia
*N*	84	974
Female gender	40 (48)	455 (47)
Age (months)	37 (25 to 49)	37 (19 to 49)
Hemoglobin (g/dL)	9.35 (8.1 to 10.4)	9.9 (8.8 to 10.9)
Weight-for-height *Z*-score	−0.7 (−1.3 to 0.02)	−0.8 (−1.5 to −0.06)
Weight-for-age *Z*-score	−0.95 (−2.2 to −0.09)	−1.3 (−2.25 to −0.45)
Height-for-age *Z*-score	−1.2 (−2.2 to −0.09)	−1.5 (−2.7 to −0.3)
Mid-upper arm circumference	14.5 (13.5 to 15.5)	14.5 (13.5 to 15.0)
Wasted	9 (11)	120 (12)
Stunted	26 (31)	369 (38)
Underweight	23 (27)	299 (31)

All continuous variables are expressed as median (interquartile range); all dichotomous variables are expressed as *N* (%).

**Table 2:** Cross-sectional relationship between nutritional status indicators and malaria parasitemia at each study collection time point

	Azithromycin communities		Placebo communities		
	Mean difference (95% CI)	*P*-value	Mean difference (95% CI)	*P*-value	*P* for interaction
Month 0					
WHZ	0.18 (−0.12 to 0.48)	0.24	−0.22 (−0.58 to 0.14)	0.24	0.10
HAZ	0.08 (−0.40 to 0.57)	0.75	0.38 (−0.19 to 0.98)	0.19	0.42
WAZ	0.16 (−0.20 to 0.53)	0.38	0.06 (−0.37 to 0.50)	0.78	0.73
MUAC	−0.17 (−0.50 to 0.15)	0.29	−0.20 (−0.59 to 0.20)	0.33	0.93
Month 12					
WHZ	−0.01 (−0.38 to 0.35)	0.94	0.14 (−0.17 to 0.44)	0.38	0.53
HAZ	0.14 (−0.34 to 0.62)	0.57	0.19 (−0.22 to 0.60)	0.37	0.88
WAZ	0.08 (−0.28 to 0.44)	0.66	0.19 (−0.11 to 0.50)	0.22	0.65
MUAC	0.07 (−0.28 to 0.41)	0.69	−0.07 (−0.36 to 0.21)	0.61	0.53
Month 24					
WHZ	−0.42 (−0.93 to 0.10)	0.11	0.16 (−0.26 to 0.59)	0.75	0.09
HAZ	0.37 (−0.32 to 1.05)	0.30	−0.05 (−0.62 to 0.53)	0.87	0.36
WAZ	0.06 (−0.61 to 0.49)	0.83	0.07 (−0.39 to 0.53)	0.78	0.73
MUAC	−0.44 (−0.99 to 0.12)	0.12	0.14 (−0.32 to 0.60)	0.56	0.12

HAZ = height-for-age *Z*-score; MUAC = mid-upper arm circumference; WAZ = weight-for-age *Z*-score; WHZ = weight-for-height *Z*-score; all models adjusted for the child’s age in months, gender, and study arm with standard errors adjusted for clustering at the community level and an interaction term for malaria parasitemia by the community treatment arm.

In models adjusted for age, gender, and study arm, there was no association between malaria parasitemia and WHZ, HAZ, WAZ, or MUAC at months 0, 12, or 24 in either placebo or azithromycin communities (Table 2). At 24 months, the direction of the association between WHZ and MUAC and malaria parasitemia was opposite in azithromycin and placebo communities, with a lower mean WHZ score in children with malaria parasitemia in azithromycin communities. However, the association was not statistically significant in azithromycin and placebo communities, and the interaction terms were not statistically significant. Results of the association between malaria parasitemia and dichotomous indicators of nutritional status were broadly consistent with results treating nutritional status as a continuous variable (Table 3). At 24 months in placebo communities, children with malaria parasitemia were more often stunted (odds ratio: 2.40, 95% CI: 1.04–5.40, *P* = 0.04); however, the CI was wide.

**Table 3:** Cross-sectional relationship between wasting, stunting, and underweight and malaria parasitemia at each study collection time point

	Azithromycin communities		Placebo communities		
	OR (95% CI)	*P*-value	OR (95% CI)	*P*-value	*P* for interaction
Month 0					
Wasted*	0.70 (0.20–1.89)	0.52	1.38 (0.44–3.63)	0.54	0.37
Stunted†	0.90 (0.47–1.68)	0.75	0.66 (0.27–1.48)	0.33	0.56
Underweight‡	0.84 (0.41–1.62)	0.61	1.15 (0.47–2.60)	0.74	0.56
Month 12					
Wasted*	0.76 (0.28–1.90)	0.57	1.17 (0.51–2.52)	0.69	0.49
Stunted†	1.15 (0.54–2.31)	0.70	0.75 (0.39–1.36)	0.36	0.37
Underweight‡	1.35 (0.62–2.79)	0.43	0.94 (0.43–1.92)	0.86	0.49
Month 24					
Wasted*	1.66 (0.24–6.99)	0.53	0.30 (0.02–1.51)	0.24	0.18
Stunted†	0.78 (0.22–2.27)	0.68	2.40 (1.04–5.40)	0.04	0.12
Underweight‡	1.03 (0.23–3.29)	0.97	0.36 (0.06–1.29)	0.18	0.29

OR = odds ratio; all models adjusted for the child’s age in months, gender, and study arm with standard errors adjusted for clustering at the community level and an interaction term for malaria parasitemia by community treatment arm.

* Defined as weight-for-height *Z*-score < −2 SD.

† Defined as height-for-age *Z*-score < −2 SD.

‡ Defined as weight-for-age *Z*-score < −2 SD from 2006 WHO guidelines.

## DISCUSSION

We were unable to find evidence of a relationship between malaria parasitemia and nutritional status in this population-based sample of children in Niger, and no evidence of a difference in the relationship in communities receiving or not receiving azithromycin after 12 or 24 months of biannual distributions. In the placebo communities, children with malaria parasitemia were more often stunted; however, the CIs were wide, and this finding could have been due to the chance given the number of comparisons in this analysis. Overall, the prevalence of malaria parasitemia was relatively low, likely because of the timing of data collection during the low transmission season. These data do not provide evidence that children with parasitemia are more likely to have worse nutritional indicators during the non-malaria and non-lean season in a region with highly seasonal malaria transmission.

In some populations, antibiotics have been shown to cause weight gain in children.^17^ However, an effect on anthropometric outcomes has not been consistently shown in studies of mass azithromycin distribution for trachoma control.^18,19^ Azithromycin has some activity against malaria and has been shown in some studies of mass distribution for trachoma to affect parasitemia and splenomegaly.^10,12,20^ In the same study in which communities were measured at the same time points, azithromycin was effective at reducing the burden of malaria parasitemia. Azithromycin distribution could hypothetically affect the relationship between malaria parasitemia and nutritional status if it affected either condition individually. However, in this study, we saw no evidence of a relationship between parasitemia and nutritional status in either azithromycin or placebo communities.

This study must be considered in the context of several limitations. First, this study included cross-sectional assessments of malaria parasitemia and nutritional status. Although a subset of children who were randomly selected for evaluation at multiple time points were followed longitudinally, the small sample size of this cohort precluded longitudinal analysis. Furthermore, samples were taken every 12 months. The presence of malaria parasitemia may not be expected to be associated with nutritional status 12 months later, and thus we did not attempt to conduct longitudinal analyses. We are, therefore, unable to comment on whether past infection or nutritional status influences current disease status. More granular measurements and larger sample sizes would be required for meaningful longitudinal analysis of this relationship. Given the highly seasonal nature of malaria and nutritional status in Niger, the results of this study are likely not generalizable to the malaria season or to settings without seasonal transmission.

In conclusion, we were unable to find evidence supporting a relationship between malaria parasitemia and nutritional status in children living in communities participating in a large trial of mass azithromycin distribution for prevention of child mortality during the low malaria transmission season. Both malaria and undernutrition are major contributors to childhood mortality and morbidity in Niger. This study did not provide evidence that children with malaria parasitemia have worse nutritional status during the low transmission season compared with those without parasitemia, and interventions to address one during this season may not affect the other.
